# Environmental modulators of vascular physiology and inflammation

**DOI:** 10.1113/EP092309

**Published:** 2025-05-11

**Authors:** Anusha N. Seneviratne, Anne Majumdar, Kalpana Surendranath, Mark R. Miller

**Affiliations:** ^1^ Department of Health Studies Royal Holloway University of London Egham Surrey UK; ^2^ Genome Engineering Laboratory, School of Life Sciences University of Westminster London UK; ^3^ Centre for Cardiovascular Science University of Edinburgh Edinburgh UK

**Keywords:** air pollution, cardiovascular disease, inflammation, medicinal plants, natural compounds, particulate matter

## Abstract

Environmental factors play a crucial role in modulating vascular inflammation, contributing significantly to the development of atherosclerosis and cardiovascular disease. This review synthesizes current evidence on how various environmental exposures influence vascular function and inflammation, with a focus on pollutants such as particulate matter and chemical toxins like bisphenols and per‐ and polyfluoroalkyl substances. These environmental stressors can trigger oxidative stress, chronic inflammation and vascular dysfunction, potentially accelerating the progression of atherosclerosis. We also explore the protective effects of natural compounds and exposure to green spaces in dampening inflammation and reducing cardiovascular risk. By examining the complex interplay between traditional risk factors and environmental exposures, this work highlights the need for comprehensive public health strategies that address both individual lifestyle factors and broader environmental determinants of cardiovascular health. We underscore the importance of further research to elucidate the precise cellular and molecular mechanisms by which environmental factors influence vascular function, with the aim of developing targeted interventions to mitigate their harmful effects and promote cardiovascular well‐being.

## Abstract (highlights)

**What is the topic of this review?**
  The review examines how environmental factors, particularly pollutants and natural compounds, influence vascular physiology, inflammation, and the development of atherosclerosis and cardiovascular disease.
**What advances does it highlight?**
  This review highlights advances in understanding how environmental factors, particularly pollutants and natural compounds, influence vascular physiology, inflammation and the development of atherosclerosis. We emphasize the need for comprehensive public health strategies addressing both individual lifestyle factors and broader environmental exposures to address the rise in cardiovascular morbidity and mortality.

## 1 INTRODUCTION

**FIGURE 1:**
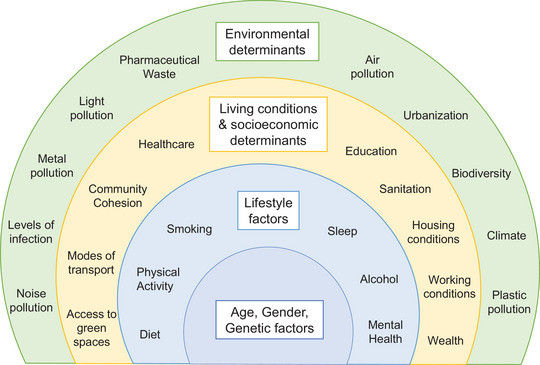
Summary of determinants of cardiovascular health.

Inflammation is a critical physiological response to injury and infection, but chronic inflammation is a key contributor to vascular diseases. One of the most common vascular pathologies is atherosclerosis, which underlies the majority of cardiovascular conditions. Atherosclerosis is the formation of lipid‐rich inflammatory plaques in the arterial intima overladen by a smooth muscle cell (SMC)‐rich fibrous cap. Rupture of this cap can expose the thrombogenic necrotic core increasing the risk of myocardial infarction and stroke. Environmental factors play a significant role in the development of atherosclerosis and the risk of cardiovascular complications of atherothrombotic diseases which can be fatal. Lifestyle factors have long been recognised as initiators of vascular dysfunction and the development of atherosclerosis including high saturated fat intake, smoking and lack of physical activity. However, the environment we live in today exposes us to a multitude of additional factors which can profoundly affect physiological function (Figure 1).

Environmental exposures that are linked to cardiovascular disease include air, water and soil pollution, noise and light pollution (potentially via disturbances of circadian rhythms), and extreme temperatures, which are an increasing threat due to climate change. Air pollution is the second leading risk factor for death and strongly associated with cardiovascular disease. Nearly 70% of the mortality linked to air pollution is caused by cardiovascular disease (ischaemic heart disease and stroke), translating to approximately 2.8 million deaths in 2019 (Miller et al., 2024a). Indeed, several air pollutants have been associated with a wide range of cardiovascular diseases, including coronary artery disease, cardiac arrest, heart failure, arrhythmia, peripheral artery disease and many others (Miller & Newby, 2019; Miller et al., 2024b). Similarly, chemical toxins polluting water and soil such as heavy metals, bisphenols, perfluoroalkyl and polyfluoroalkyl substances (PFAS), and more recently, microplastics and nanoplastics, are all associated with increased cardiovascular risk (Miller et al., 2024c; Münzel et al., 2025). Many of these factors are linked to increased oxidative stress and inflammation, which could play a greater pathogenic role in many diseases than is currently appreciated (Figure 1).

In support of a key role for inflammation, some natural interventions can dampen the inflammation associated with vascular disease. Dietary constituents such as sulforaphane and curcumin reduce vascular inflammation and cardiovascular risk and exposure to green spaces can also mediate these effects. Natural interventions focusing on dietary and lifestyle changes could be promising alternatives to pharmacological approaches in reducing vascular dysfunction and cardiovascular disease. This is of particular relevance to our ageing population where adverse reactions from polypharmacy are increasing.

Despite the wealth of evidence linking man‐made pollutants to vascular disease, the precise cellular and molecular mechanisms of disease are yet to be fully established. A better understanding of the key role of systemic inflammation in the cardiovascular effects of pollution may offer valuable insights to guide public health guidance and interventions. This review will briefly address our current understanding of vascular dysfunction, and the role of inflammation in the development of atherosclerosis and subsequent cardiovascular disease. We synthesise the current evidence on how air particulates and associated chemicals influence vascular function and inflammation, highlighting gaps in our understanding of the underlying mechanisms. We explore the beneficial interventions to vascular function, which are necessary to develop comprehensive public health strategies to mitigate against and adapt to detrimental environmental exposures.

## 2 VASCULAR DYSFUNCTION AND ATHEROSCLEROSIS

The primary cause of coronary and cerebrovascular disease is atherosclerosis (Herrington et al., 2016). Endothelial dysfunction is an early feature of the initiation of atherosclerosis, resulting from a response to injury by noxious substances such as cigarette smoke, biomechanical injury resulting from turbulent blood flow, or various stressors linked to conditions such as diabetes or hypertension.

**FIGURE 2:**
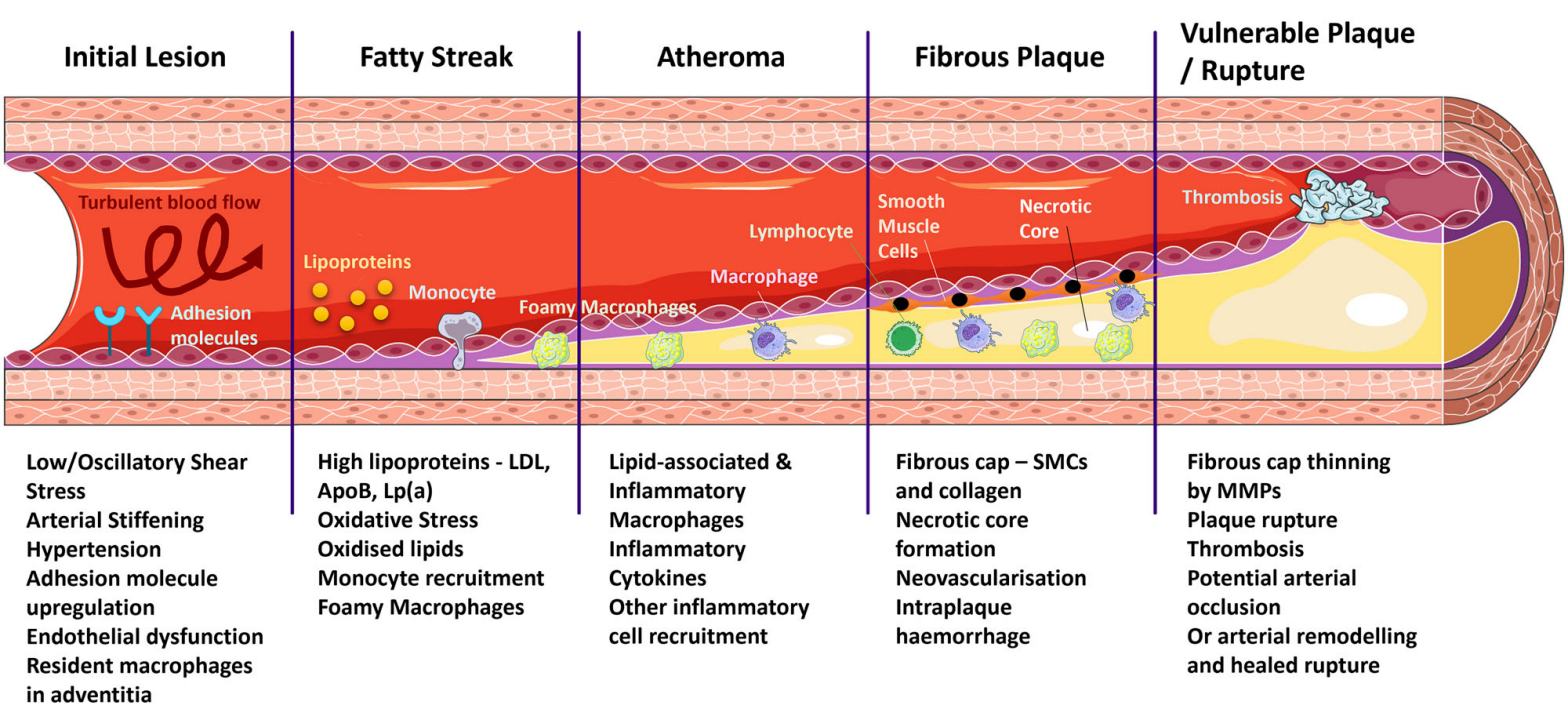
Stages of atherosclerotic plaque development from left to right. ApoB, apolipoprotein B**;** LDL, low density lipoprotein; Lp(a), lipoprotein(a); MMP, matrix metalloproteinase; SMC, smooth muscle cells.

Lipid accumulation in the vessel wall is a key feature of atherosclerosis, a process that is driven by inflammation, primarily dictated by macrophages, at all stages of disease (Figure 2). Elevated plasma low‐density lipoprotein (LDL) concentrations lead to LDL transport through the endothelium and into the intima (Nielsen, 1996). Apolipoprotein B (ApoB) content of LDL particles plays a central role in the progression of atherosclerosis (Glavinovic et al., 2022). Lipoprotein(a) (Lp(a)) is an apoB100‐containing lipoprotein, primarily genetically determined and a causal risk factor for atherosclerotic cardiovascular disease (Reyes‐Soffer et al., 2022). Lp(a) can contribute to cholesterol deposition in the intima (Qin et al., 2024).

In contrast, high density lipoprotein (HDL) cholesterol is inversely related to atherosclerosis, as it facilitates reverse cholesterol transport from plaque macrophages to the liver for excretion (Rosenson et al., 2012) and has anti‐inflammatory actions (De Nardo et al., 2014).

Oxidative stress leads to endothelial dysfunction, the activation of inflammatory pathways and the proliferation of vascular smooth muscle cells (SMC). Minimally modified LDL (mmLDL) is oxidised by reactive oxygen species (ROS), myeloperoxidase, nitric oxide (NO) and 15‐lipoxygenase, for example, to form oxidised LDL (oxLDL) in the intima (Glass & Witztum, 2001). Oxidative stress and inflammation often occur in tandem, with the effects of one promoting or amplifying the other, exacerbating atherosclerosis in different stages of disease progression.

Pro‐inflammatory cytokines and oxLDL upregulate endothelial cell expression of selectins and adhesion molecules facilitating monocyte entry into the intima (Hansson & Libby, 2006); macrophages phagocytose modified LDL; and the accumulation of foam cells on the intimal surface forms ‘fatty streaks’ (or ‘intimal xanthomatas’), the earliest form of atherosclerotic plaque (Virmani et al., 2000) (Figure 2).

Macrophages infiltrate the lipid pool and transform into foam cells; they can apoptose leading to the release of free cholesterol, causing a lipid‐rich necrotic core to develop over time (Figure 2). Thrombogenic material in the plaque is introduced by intraplaque haemorrhage due to ruptured neovessels and the necrotic core. The formation of a thick fibrous cap over the core can maintain plaque integrity and this avoids contact of the intraplaque pro‐thrombotic tissue factor‐rich necrotic core with flowing blood (Hansson & Libby, 2006). Thinning and rupture of the fibrous cap is associated with the presence of matrix metalloproteinases (MMPs) (Figure 2).

The recent introduction of single‐cell technologies allowing high‐dimensional analysis of cell populations within atherosclerotic plaques has demonstrated the heterogeneity of macrophage phenotypes, which are far more complex that the previous traditional dichotomy of M1 and M2 macrophages. These macrophage subsets can be broadly categorised into three main types – resident macrophages in the adventitia (Park et al., 2022), inflammatory macrophages in the intima (Park et al., 2022; Seneviratne et al., 2017), and lipid‐associated macrophages also in the intima (Dib et al., 2023). However, all macrophage subsets show plasticity in adapting to their microenvironment and adopt a variety of phenotypes, many of which are yet to be fully characterised (Zernecke et al., 2020).

The links between cardiovascular disease and lifestyle factors such as Westernized high‐fat diet, salt and sugar intake, smoking, excess alcohol consumption and lack of physical activity have been well established for decades. Poor lifestyle factors often first lead to secondary risk factors such as central obesity, dyslipidaemia, hypertension and insulin resistance. Due to the frequent clustering of these risk factors, they have been generically classified as metabolic syndrome. Dyslipidaemia and diets high in saturated fat have long been a focus of research into atherogenesis. Accumulation of lipids in the arterial intima usually initiates atherogenesis in arterial regions exposed to slow blood flow causing low shear stress at curvatures and turbulent blood flow causing oscillatory shear stress at branch points in large arteries (Nakashima et al., 1994), which is also associated with endothelial dysfunction (Bonetti et al., 2003). However, the development of endothelial dysfunction cannot only be explained by exposure to slow or turbulent blood flow and may involve exposure to other environmental factors.

## 3 THE ENVIRONMENT AND CARDIOVASCULAR DISEASE

To address the gaps in understanding the pathology of vascular dysfunction and atherosclerosis, we may need to look beyond the traditionally considered risk factors. Inflammation plays an important role in the development of atherosclerosis. This has been observed over human history as our species endured many inflammatory insults which we now know are closely associated with vascular dysfunction and atherosclerosis. To illustrate this, significant levels of atherosclerosis were detected from computed tomography scans of mummies from four distinct geographical regions spanning a period of more than 4000 years (Thompson et al., 2013). This could reflect different exposures triggering inflammation over time such as consumption of saturated fat among hunter–gatherers, smoke inhalation from cooking over wood fires and high levels of chronic infection in premodern conditions (Thompson et al., 2013).

Today humans are exposed to a multitude of stresses, many of which are associated with increased inflammation and the incidence of many inflammatory diseases. We can consider these factors in an adapted version of the Dahlgren–Whitehead rainbow model (Dahlgren & Whitehead, 2021), originally used to describe determinants of health (Figure 2). Most health determinants outlined in the figure are closely linked to the incidence of cardiovascular disease. Vascular disease typically becomes more apparent at older age, and more so in males. Genetic factors such as familial hypercholesterolaemia greatly increase the risk of atherosclerosis. Individual lifestyle factors and poor living and socioeconomic conditions are known to drive chronic stress and poor mental health, which can increase blood pressure, vascular dysfunction and chronic low‐grade inflammation. Vascular compliance, or the ability of blood vessels to expand and contract in response to changes in blood flow and pressure, has long been regarded as a useful indicator of arterial health and disease. As we age, vessels stiffen resulting in a loss of arterial compliance, which can result in hypertension and high pulse pressure (Glasser & Dudenbostel, 2011). Hypertension can induce arterial stiffening by damaging endothelial cells, triggering endothelial dysfunction and subsequent vascular remodelling (Tedgui & Mallat, 2004). A vicious cycle thus ensues, whereby hypertension, endothelial dysfunction and arterial stiffening exacerbate each other, leading to inflammation of the vascular wall and promoting the development of atherosclerosis (Figure 2).

We are increasingly becoming aware of environmental exposures that are associated with these pathogenic processes (Figure 1). Light and noise pollution, often from increasing urbanisation, can cause lack of sleep, increased stress and cortisol production (Barbaresco et al., 2019). Climate change increases the frequency of extreme weather events which can directly harm the heart and vascular system, increasing the risk of heart failure particularly in vulnerable patients and the elderly, for example (Sliwa et al., 2024). Biodiversity loss and deforestation is accelerating climate change while exacerbating the risk of zoonotic infections and pandemics, which can increase morbidity in the population. We are increasingly exposed to many pollutants due to increasing urbanisation and industrialisation, and notably a number of these are linked to our continued reliance on fossil fuels. The WHO regional office for Europe recently highlighted four major commercial products – alcohol, tobacco, processed food and beverages, and fossil fuels – as causing an estimated 2.7 million deaths annually in the WHO European Region from non‐communicable diseases, including cardiovascular disease (Kasturiarachchi et al., 2024).

## 4 POLLUTION AND THE VASCULATURE

There is increasing concern about the many pollutants that we are exposed to, not just from the air, but also from water, food and soil. Ambient air pollution consists of gases, such as nitrogen dioxide (NO_2_), sulphur dioxide (SO_2_), ozone (O_3_) and carbon monoxide (CO), and particulate matter (PM). Air pollution is linked to almost 7 million deaths globally every year (Miller et al., 2024a). Sources of these pollutants include combustion, vehicles, industry, wildfires, domestic heating, cooking, mining and agriculture. Airborne microplastics shed from synthetic textiles and plastic items are also found in PM (Thompson et al., 2024). These pollutants are all associated with adverse health effects, the toxicity of which is dependent on a range of physicochemical properties of the pollutant. In relation to air pollution, while both gaseous and particulate pollution can have detrimental effects on health, more research has focused on PM as the health associations observed appear to be greatest and most consistent for these constituents, especially in relation to cardiovascular disease. Multiple epidemiological studies have found associations between air pollution and atherosclerosis (Hahad et al., 2025; Künzli et al., 2011), which has been accompanied by a growing evidence base on underlying mechanisms.

### 4.1 Gaseous pollutants

Some studies suggest gases from vehicle emissions can have significant cardiovascular effects, even in the absence of PM. Exposure to petrol emissions, which includes PM, CO and NO_2_ increases the expression of MMP‐3, MMP‐7 and MMP‐9, tissue inhibitor of metalloproteinases‐2 (TIMP‐2), endothelin‐1 in the arteries of ApoE^−/−^ mice, as well as haem oxygenase‐1, nitrotyrosine and lipid peroxides as markers of oxidative stress (Lund et al., 2007), which can also be activated by intraplaque haemorrhage (Boyle et al., 2011). Similar changes occurred when PM was filtered out suggesting the gaseous components of exhaust fumes were also increasing markers of vascular oxidative stress and remodelling (Lund et al., 2007). Exposure of ApoE^−/−^ mice to diesel emissions, with PM filtered out, alters the expression of markers of vascular remodelling such as MMP‐9, and lipid peroxidation in the aorta (Campen et al., 2010). Human coronary artery endothelial cells increase expression of intercellular adhesion molecule 1 (ICAM‐1) and vascular cell adhesion molecule 1 (VCAM‐1) when incubated with plasma from human subjects exposed to NO_2_ (Channell et al., 2012).

Exposure of rats to ozone increases markers of arterial injury such as oxidative stress markers, thrombotic factors (tissue factor, plasminogen activator inhibitor‐1, tissue plasminogen activator and von Willebrand factor), MMP‐2, MMP‐3 and TIMP‐2, and vasoconstriction (Kodavanti et al., 2011). Endothelial dysfunction and impaired vasodilatation in mice exposed to ozone is dependent on the scavenger receptor CD36 (Robertson et al., 2013).

### 4.2 Particulate matter

PM can be classified by diameter: PM10 are less than 10 µm wide, PM2.5 are fine particles less than 2.5 µm, and PM0.1 are ultrafine particles smaller than 100 nm. Both PM10 and PM2.5 are routinely measured by networks of ground monitoring stations the world over and are frequently employed in epidemiological studies. PM2.5 are the most studied category since it is more consistently linked to health outcomes. PM2.5 exposure has been linked to vascular dysfunction (Møller et al., 2011), atherosclerosis (Künzli et al., 2010) and myocardial infarction (Peters et al., 2001). Animal studies have shown that inhalation of PM2.5 promotes the development of atherosclerosis (Miller et al., 2013). Smaller ultrafine particulates (PM0.1) have been linked to significant detrimental effects on the vasculature (Araujo et al., 2008) such as arterial vasoconstriction (Brook et al., 2002). Animal studies have shown that diesel exhaust particles (DEP), an ultrafine PM‐rich air pollutant, promote the development of atherosclerotic plaques, both in terms of size and complexity, which may reflect plaque vulnerability (Miller et al., 2013). While epidemiological studies of PM0.1 are limited due to the difficulties of measuring these particles widely in the real world, surrogates of PM0.1 have been associated with cardiovascular outcomes (Chen et al., 2020). The high surface area of PM0.1 compared to its mass, as well as the complex composition of many sources of PM0.1 in urban air pollution, suggests these particles could be especially harmful, and this has been supported by toxicological studies (Miller & Newby, 2019; Stone et al., 2017). Additionally, nanosized particles can cross from the lung into the bloodstream and accumulate in atherosclerotic plaques (Miller et al., 2017).

Most mechanistic studies have so far focused on the promotion of oxidative stress, which, as previously mentioned, can lead to increased endothelial cell expression of adhesion molecules, facilitating leukocyte entry into the intima, and the induction of specific inflammatory pathways (Batty et al., 2022). PM2.5 exposure triggers systemic inflammation and oxidative stress (Kampfrath et al., 2011). PM2.5 can contribute to oxidative stress by increasing the formation of ROS and reactive nitrogen species. Rat aortic rings exposed to DEP generated oxygen‐centred free radicals which scavenge vascular NO resulting in impaired vascular relaxation (Miller et al., 2009). The ROS generated from complex particles like DEP will be a product of both the innate capacity of the particulates to generate free radicals and their ability to stimulate cellular enzymes that can produce free radicals, for example, NADPH oxidase (Miller et al., 2012). Different chemical species in DEP will instigate these pathways and specific free radicals to different extents, but the adsorbed organic compounds, such as polyaromatic hydrocarbons, quinones and redox‐active metals, are believed to play a significant role (Romieu et al., 2007). Oxidative stress increases the formation of oxLDL, which accumulate in the vessel wall, contributing to plaque progression (Liu et al., 2019). PM2.5 also increases mitochondrial damage in macrophages, and when combined with increased oxLDL accumulation, results in the apoptosis of macrophage foam cells (Liu et al., 2019), which can add to the formation of the necrotic core of an advanced atherosclerotic plaque.

**FIGURE 3:**
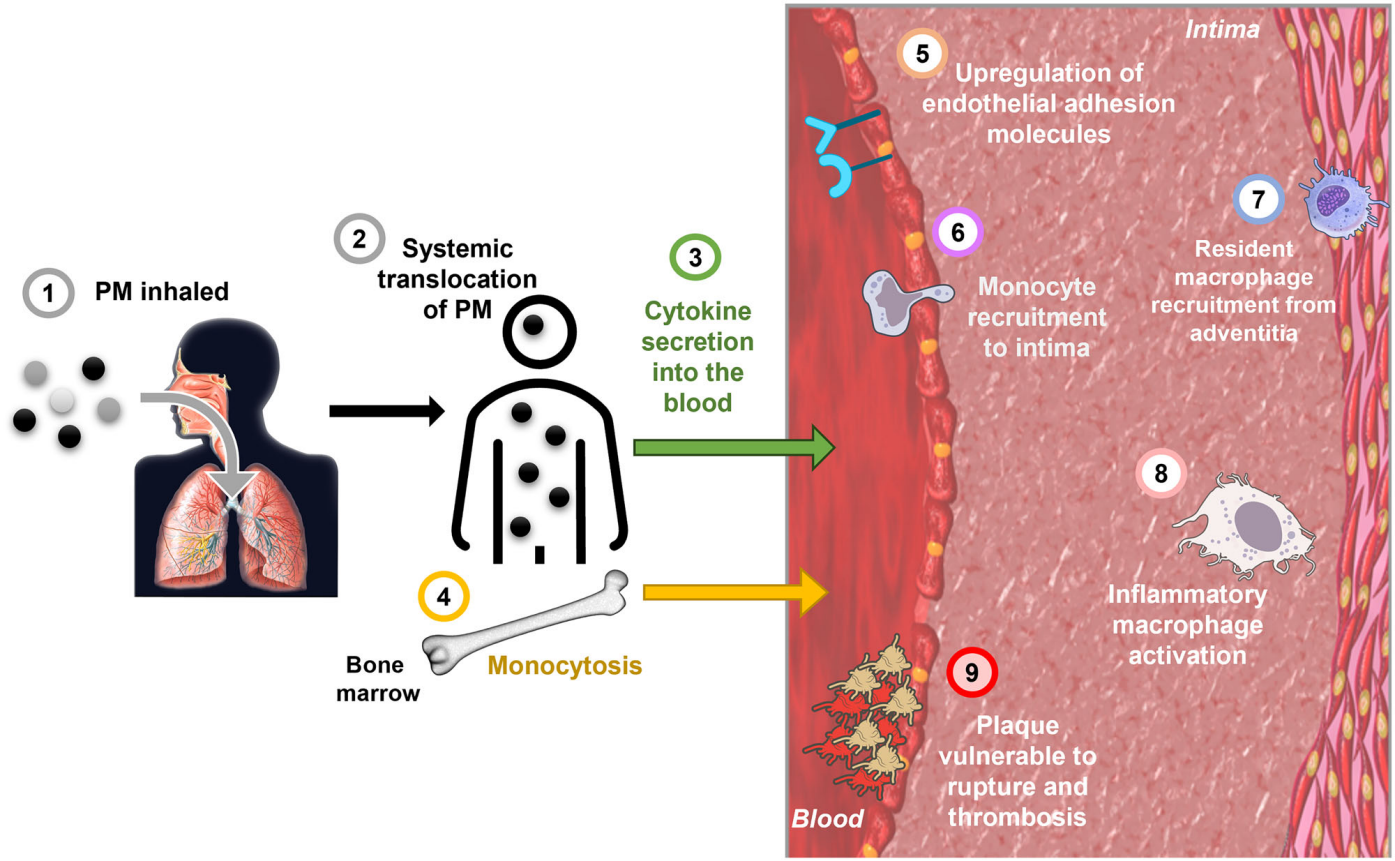
Potential mechanisms by which pollutants such as particulate matter cause vascular dysfunction and promote atherosclerosis.

More broadly, there is evidence that PM10 can increase circulating cytokines (Figure 3) such as interleukin (IL)‐1β, IL‐6 and granulocyte–macrophage colony‐stimulating factor in humans (Van Eeden et al., 2001). In mice, exposure to PM2.5 increases circulating levels of tumour necrosis factor (TNF)‐α, monocyte chemoattractant protein (MCP)‐1 and the IL‐12 p70 subunit, while the anti‐inflammatory cytokine IL‐10 is decreased (Kampfrath et al., 2011). These changes in circulating cytokines are dependent on the expression of Toll‐like receptor (TLR)‐4 (Kampfrath et al., 2011). TNF‐α (Bai & Van Eeden, 2013) and IL‐6 are elevated in response to airborne particles, including DEP (Terashima et al., 2012), and both are known to promote monocyte chemotaxis and increase cardiovascular risk.

It is not clear which cells are the primary source of these upregulated inflammatory cytokines in the vasculature. Resident immune cells in the lung that are directly exposed to particulates could secrete these cytokines, which are subsequently transported into the bloodstream via the respiratory membrane. DEP stimulates the secretion of inflammatory factors from bronchial epithelial cells and alveolar macrophages in vitro (Boland et al., 2000), suggesting they are potential sources of inflammatory mediators in the lung and circulation. If significant levels of particulates enter the bloodstream, these could directly activate circulating leukocytes and cells of the vascular wall such as endothelial cells to generate inflammatory cytokines (Figure 3). For example, in vitro studies show PM10 induces IL‐1β (Marín‐Palma et al., 2023) secretion by healthy peripheral blood mononuclear cells (PBMCs). Exposure to air pollution is associated with health conditions in many different organ systems (Miller et al., 2024a). If particulates are trafficking to these organs, resident immune cells could be stimulated to produce inflammatory cytokines contributing to systemic inflammation. Once in the vasculature, pro‐inflammatory cytokines promote vascular inflammation, playing a major role in the development of atherosclerosis (Tedgui & Mallat, 2006).

PM exposure is linked to increased circulating leukocytes and monocytosis (Goto et al., 2012a). Short‐term exposure of adult males to PM2.5, PM10, NO_2_ and CO in Beijing was associated with increased neutrophil, lymphocyte and monocyte counts in their peripheral blood (Xue et al., 2023). Instillation of ambient PM10, or supernatants of alveolar macrophages incubated with PM10 in vitro, into the lungs of rabbits triggered an increase in circulating immature granulocytes and accelerated monocyte release from the bone marrow (Goto et al., 2012b) suggesting cytokines originating in the lung are capable of triggering monocytosis. PM2.5 induced monocyte mobilization from the bone marrow in a TLR4‐dependent manner in mice (Kampfrath et al., 2011).

Endothelial cell dysfunction may assist monocyte infiltration into the intima (Figure 3). PM can activate endothelial cells, leading to the upregulation of adhesion molecules such as ICAM‐1 (Yatera et al., 2008) and VCAM‐1 (Pope et al., 2016). Both adhesion molecules play a key role in the development of early atherosclerotic lesions (Cybulsky et al., 2001), enabling monocytes to adhere to endothelial cells and translocate into the intima. Exposure to PM2.5 is associated with endothelial injury and endothelial cell apoptosis, indicated by an increase in circulating endothelial microparticles, soluble adhesion molecules and aggregates of platelets with monocytes (Pope et al., 2016). These responses would increase the likelihood of atherogenesis being initiated, promoting monocyte recruitment to the intima, and potentially increase the risk of thrombosis at sites of plaque erosion or rupture.

Monocytosis could be a key mechanism of PM‐induced atherosclerosis progression as macrophages within atherosclerotic lesions are known to be largely derived from increases in circulating monocytes (Swirski et al., 2007) originating from the bone marrow (Figure 3) (Williams et al., 2020). Macrophages can also be derived from vascular SMCs (Allahverdian et al., 2014) or resident arterial macrophages of embryonic origin (Ensan et al., 2016), but the majority are derived from circulating monocytes. There is evidence that macrophages can proliferate locally once established in the plaque (Robbins et al., 2013) although it is not known if PM or other pollutants can affect this dynamic. In addition to increased circulating monocytes, there is evidence for an increase in peripheral blood in response to PM2.5 of CD4^+^ and CD8^+^ T cells (Pope et al., 2016), which make up a high proportion of the immune cell population in human plaques (Edsfeldt et al., 2022).

In vitro studies have demonstrated that PM10 and PM2.5 activate TLR‐2 and ‐4 (Cevallos et al., 2017), both of which are pro‐atherogenic when chronically activated (Michelsen et al., 2004; Monaco et al., 2009). The NLRP3 inflammasome and downstream cytokine IL‐1β are also activated by PM10, PM2.5 (Cevallos et al., 2017) and DEP (Provoost et al., 2011). With many in vitro studies demonstrating inflammatory activation of macrophages and cytokine production (Miyata & van Eeden, 2011), this is most likely a key mechanism in the progression of atherosclerosis in response to PM. Further research is needed to more precisely determine how macrophage heterogeneity is impacted by environmental exposures and whether other pollutants skew macrophages towards inflammatory phenotypes.

A key determinant of the risk of cardiovascular events is destabilisation of atherosclerotic plaques. Exposure to high levels of PM2.5 is more likely to be associated with the presence of thin‐cap fibroatheromas, in patients experiencing acute coronary syndrome (Montone et al., 2022). In addition, increased exposure to PM2.5 is associated with a greater risk of new plaques formed with either a fibrofatty or necrotic core (Yang et al., 2019), both considered key features of vulnerable plaques. In ApoE^−/−^ mice, exposure to DEP is associated with larger plaques with buried fibrous layers, which could potentially represent plaques that have undergone repeated cycles of rupture and repair (Miller et al., 2013).

Overall, it is now well established that air pollution can promote atherosclerosis through a range of different mechanisms including oxidative stress, inflammation, endothelial dysfunction and by facilitating lipid accumulation. While this mechanistic evidence presents a compelling case for the causality of epidemiological associations between PM and atherosclerosis, further elucidation of the nuances of the mechanisms identified, as well as the ability of well‐explored types of PM to promote atherosclerosis, would help further establish the risks to vulnerable and susceptible individuals.

### 4.3 Chemical pollution

Chemicals, and potentially particles carrying these chemicals, which have been found to contaminate water and soil can promote cardiovascular risk (Münzel et al., 2024). Bisphenol A (BPA) exposure increases the progression of atherosclerosis in rabbit models of disease, accompanied by increased lipid accumulation, endoplasmic reticulum stress and upregulation of inflammatory markers in the liver and serum (Fang et al., 2014). BPA exposure also accelerates atherosclerosis progression in high‐fat‐fed ApoE^−/−^ mice (Kim et al., 2014). These findings are supported by human studies where urinary BPA concentrations were positively correlated with the incidence of coronary artery disease (Melzer et al., 2012). BPA and phthalates found in the circulation of elderly patients also correlated with atherosclerosis of the carotid artery (Lind & Lind, 2011). Despite efforts to replace BPA with similar compounds such as bisphenol S (BPS), these structural analogues continue to exhibit harmful properties as they are associated with an increased risk of coronary heart disease in elderly patients, hypertension, myocardial infarction and stroke (Wang et al., 2022). Furthermore, bisphenol F and BPS, are associated with chronic kidney disease and congestive heart failure (Lu et al., 2023).

Another group of endocrine‐disrupting chemicals are PFAS, which are also linked to hypertension and dyslipidaemia, both important risk factors for cardiovascular disease (Schlezinger & Gokce, 2024). Per‐ and poly‐fluoroalkyl substances like perfluorooctanoic acid are linked to the risk of cardiovascular disease, peripheral artery disease (Shankar et al., 2012) and stroke (Simpson et al., 2013).

Bisphenols and PFAS are often used as plastic additives. Microplastics and nanoplastics derived from plastics such as polyethylene and polyvinyl chloride have been found within macrophages in human atheromas, and their presence was associated with a greater risk of myocardial infarction or stroke (Marfella et al., 2024). Polystyrene nanoplastics increase oxidative stress in endothelial cells by inducing NADPH oxidases and ROS formation resulting in premature senescence of endothelial cells and endothelial dysfunction (Shiwakoti et al., 2022). It should be noted that there are multiple routes for the human population to be exposed to plastic pollution, such as air pollution, occupational exposure, food and water contamination, and therefore there is a need to establish which routes of exposure present the greatest risk (Landrigan et al., 2023). Research is urgently needed on the health impacts and mechanisms of plastics and the additive chemicals contained within them, particularly if they also induce systemic inflammation to an extent that increases the risk of vascular disease and other diseases with a prominent inflammatory component.

Given most people spend over 80% of their time indoors, where concentrations of airborne particles can be periodically higher than outdoors, exposure to indoor PM is of increasing concern. There has been little exploration of the impact of indoor dust in relation to cardiovascular disease. We do know, however, that dust, particularly in occupational settings such as recycling plants and textile manufacturing, can contain endocrine‐disrupting chemicals like bisphenols and PFAS, microplastic particles, as well as heavy metals like lead and cadmium (Landrigan et al., 2023). Chemicals such as polybrominated diphenyl ethers are used as flame retardants in consumer products and are detected in domestic settings, for example, leaching from furniture, released after cleaning plastic floors, and from personal care and cosmetic products (Miller et al., 2024c). The harmful effects of heavy metals, independent of other contaminants, are illustrated by studies on exposure to cigarette smoke, which is rich in metal contaminants like arsenic, cadmium, chromium, nickel and lead (Caruso et al., 2013). Heavy metals like lead, cadmium and mercury, through exposure routes such as soil, food, water, tobacco smoke, electronic cigarettes, petrol emissions, lead‐based paints and water pipes, have been associated with endothelial dysfunction and inflammation (Lamas et al., 2021). Vehicle brake abrasion PM, which contains more metals/metalloids than DEP (such as iron, zinc and copper), increases secretion of IL‐8 and TNF‐α and the anti‐inflammatory cytokine IL‐10 by macrophages in vitro, while phagocytic clearance of bacteria is diminished (Selley et al., 2020). The cardiovascular effects of non‐exhaust emissions from vehicles requires attention given the potential for heavier electric vehicles to generate higher concentrations of these PM than lighter vehicles, albeit this may be offset by improvements in regenerative braking (Fussell et al., 2022).

Lead exposure is linked to inactivation of paraoxonase activity, leading to reduced antioxidant effects of HDL (Solenkova et al., 2014) and an increase in proatherogenic ROS. Lead can also induce hypertension by acting as a calcium substitute and interacting with calmodulin during NO synthesis, leading to alterations in vascular resistance (Vaziri, 2008). Exposure of rats to lead via drinking water increases activation of the p65 subunit of nuclear factor‐kappa B (NF‐κB) in kidney tubulointerstitial cells, which is likely to contribute to hypertension (Rodríguez‐Iturbe et al., 2005). Activation of NF‐κB in endothelial cells (Hajra et al., 2000) and macrophages is known to be proatherogenic. Similarly, the metal cadmium can increase oxidative stress and activate cell apoptosis (Biagioli et al., 2008). Cadmium can cause abnormal arterial tone by reducing phosphorylation of eNOS, resulting in reduced NO production (Majumder et al., 2008). Serum cadmium levels are associated with alterations in blood triglyceride and glucose levels, and subsequently an increased risk of carotid artery atherosclerosis (Kim et al., 2022).

Studies on the impact of chemical toxins on the body are in their infancy but rapidly surging due to a greater recognition of exposure to more chemical entities and the increasing threat they pose to health at levels found in the environment.

## 5 NATURAL COMPOUNDS AND MEDICINAL PLANTS

Despite the wealth of knowledge demonstrating the harmful effects of air pollution on human health, progress to improve air quality globally has been slow. This necessitates alternative means of protecting our health from the harms of pollution as strategies to reduce emissions come into full effect. Medicinal plants are a potential option. Approximately 80% of the world's population, particularly in low‐ and middle‐income countries (LMICs), rely on traditional medicines, many of which are plant‐derived natural compounds and may, therefore, have a useful balance of efficacy compared to side effects, or represent useful adjunct therapies especially for vulnerable and elderly patients who may already be taking multiple medications and are at greater risk of adverse effects from polypharmacy. Additionally, many pharmaceutical drugs are not available to LMICs due to cost implications, and thus many already rely on natural remedies due to lower cost or being more accessible.

As many pollutants have been shown to increase oxidative stress, antioxidants could be a useful preventative measure. Ascorbic acid (vitamin C), a water‐soluble vitamin, can scavenge many free radicals and oxidants discharged from activated neutrophils and macrophages (Kelly & Mudway, 2003). The lipid‐soluble vitamin E is also capable of scavenging free radicals and disrupting lipid peroxidation (Burton & Ingold, 1981). The vitamin A precursor β‐carotene is a scavenger of ROS and helps prevent downstream inflammation (Romieu et al., 2007). However, studies of oxidative stress and inflammation in the lungs following antioxidant supplementation have generally only shown beneficial effects in vulnerable individuals suffering from asthma (Mudway et al., 2006). *N*‐Acetylcysteine reduces ROS, TNF‐α and IL‐6 production by human endothelial tube cells exposed to DEP (Tseng et al., 2015).

However, the promising epidemiological evidence relating to antioxidants in vascular disease has not been endorsed by subsequent human trials, which, for example, indicated that vitamin E supplementation actually increased risk of death. Clinical guidelines for cardiovascular disease prevention and management therefore focus on antioxidants forming a part of a healthy, balanced diet rather than supplementation, with at least five portions of fruit and vegetables per day (Deanfield et al., 2014). Some speculation on limitations in application of antioxidant supplementation is around their short lifespan and hence the need to have them present at times that coincide with high oxidant stress to be beneficial. Therefore, the route of administration could be key to the effectiveness of antioxidants. Inhalation of antioxidants, which increases their availability in the airways, could provide greater protection against oxidative damage and downstream inflammation (Schichlein et al., 2023). For example, vitamin D nebulisation to the lungs of mice reduced cytokine and chemokine expression and leukocyte infiltration (Serré et al., 2022). Intranasal delivery of antioxidant oils including vitamin E to humans induced the expression of antioxidant genes such as *Nrf2* while dampening pro‐inflammatory signalling (Gao et al., 2011). Nrf2 activation in macrophages is atheroprotective through the upregulation of haem oxygenase‐1 and IL‐10 expression, which can occur in response to intraplaque haemorrhage, while oxidative stress is suppressed (Boyle et al., 2011).

Some exposures of natural origin can dampen inflammation associated with vascular disease. Sulforaphane, the active compound in broccoli, can act as an antioxidant and prevent inflammatory activation of endothelial cells in sites susceptible to atherosclerosis by activating Nrf2, with subsequent expression of VCAM‐1 on the cell surface (Zakkar et al., 2009). Thymoquinone, a constituent of *Nigella sativa* seeds, reduces leukocytosis, IL‐6 production, oxidative stress and pro‐thrombotic markers in mice exposed to DEP (Nemmar et al., 2011).

Curcumin, the active compound in turmeric, has long been used to treat skin, hepatic and digestive conditions in Asia (Toda et al., 1985). Curcumin inhibited TLR4 expression in ApoE^−/−^ mice as well as the expression of proatherogenic markers such as IL‐1β, TNF‐α, NF‐κB, VCAM‐1 and ICAM‐1 in the aorta, which correlated with reduced atherosclerosis (Zhang et al., 2018). in vitro studies show curcumin can reduce the harmful effects of PM2.5 on human microvascular endothelial cells by attenuating the induction of oxidative stress, and expression of TNF‑α, IL‑8, NF‐κB, VCAM‐1 and ICAM‐1 (Shi et al., 2017). Curcumin pre‐treatment of mice prior to DEP exposure prevents increases in systolic blood pressure, C‐reactive protein, TNF‐α and pro‐thrombotic factors in the circulation (Nemmar et al., 2012).

We have previously shown that the French lilac plant‐derived drug metformin – typically used to treat type 2 diabetes – reduces the progression of atherosclerosis by activating the anti‐inflammatory AMP kinase (AMPK) β subunit and subsequent phosphorylation of activating transcription factor 1 in macrophages (Seneviratne et al., 2021). Metformin is known to be a highly safe drug with few side effects compared to other medications. Metformin can reduce levels of oxidative stress and cell death induced by PM2.5, resulting in some protection from lung dysfunction (Gao et al., 2020). Treating mouse and human alveolar macrophages with metformin following PM2.5 exposure prevents the generation of mitochondrial ROS, IL‐6 release and arterial thrombosis (Soberanes et al., 2019), by inhibiting TLR4 signalling, MyD88 and NF‐κB activation (Shi et al., 2020).

Exposure to green spaces can reduce vascular inflammation and cardiovascular risk. This could be through positive effects on mental health which reduce stress and levels of the cortisol hormone that are associated with hypertension and inflammation. Forest trees release a variety of biogenic volatile organic compounds (BVOCs), the majority of which are terpenes and terpenoids, to repel herbivores and pathogenic microorganisms (Unsicker et al., 2009). Evidence from in vitro and in vivo studies suggest volatile terpenes and terpenoids can exhibit anti‐inflammatory effects. In a variety of inflammatory disease models, such as lung injury and arthritis, compounds such as d‐limonene and camphor can reduce oxidative stress, inflammatory cytokine production, inflammasome and NF‐κB activation (Kim et al., 2020). While studies on cardiovascular disease models are limited, there is some evidence, for example, that l‐limonene can improve lipid profiles, reduce oxidative stress and inflammatory markers in diabetic rats by activating AMPK and Sirtuin 1 while downregulating the NF‐κB p65 subunit in the aorta (Han et al., 2023). Combined with the ability of plants and trees to absorb high levels of pollution, green spaces are important to protect our health from the harms of pollution and mitigate against climate change.

The lack of epidemiological evidence supporting the beneficial effects of natural compounds does raise doubt about the potential efficacy of these compounds. However, while each compound in isolation may not be sufficient, a combination of many compounds introduced through diet or combined supplementation may be necessary to see significant health benefits. It could also be that potent benefits of many lesser‐known natural compounds used in traditional medicine have yet to be realised due to limited testing of traditional medicine in Western‐centric medical research. These dietary approaches should also be considered in addition to broader lifestyle changes such as limiting unhealthy foods, improved physical activity and urban landscapes that are designed to support access to green spaces. Independent of other environmental exposures, healthy diets rich in a variety of plant‐derived foods that are affordable and accessible to all should be a prominent part of strategies to reduce non‐communicable diseases.

## 6 CONCLUSIONS

Environmental factors play a significant role in modulating vascular inflammation, contributing to the development of atherosclerosis and cardiovascular disease. We have highlighted the complex interplay between traditional risk factors, such as diet and lifestyle, and environmental exposures, including air and chemical pollution. These pollutants can trigger oxidative stress, chronic inflammation and vascular dysfunction, potentially accelerating the progression of atherosclerosis. Increasing awareness of environmental determinants of cardiovascular health underscores the need for comprehensive public health strategies that address both individual lifestyle factors and broader environmental exposures. Conversely, natural compounds and exposure to green spaces show promise in dampening inflammation and reducing cardiovascular risk. This is especially important for LMICs where populations do not have sufficient access to treatments.

Vulnerable individuals living in highly polluted areas may also need to adjust their behaviour to reduce their exposure to harmful pollutants. For example, use of FFP2 face masks, rather than surgical masks, helps to reduce arterial pressure (Langrish et al., 2012). Use of air purifiers with high‐efficiency particulate air (HEPA) filtration to lower indoor exposure to PM2.5 can reduce blood pressure, MCP‐1 and IL‐1β (Chen et al., 2015). Following transport routes with lower levels of ambient air pollution (Montone et al., 2023) and active forms of travel such as walking and cycling can outweigh the harms caused by exposure to PM2.5 at the average global levels of 22 µg/m^3^. However, harms exceed the benefits when PM2.5 reaches 100 µg/m^3^ (Tainio et al., 2016).

Future research should focus on elucidating the precise cellular, molecular and immunological mechanisms and the causal sequence by which environmental factors, particularly emerging pollutants of concern, promote vascular disease, with the aim of developing targeted public health interventions to mitigate their harmful effects and promote cardiovascular health.

## AUTHOR CONTRIBUTIONS

Anusha N. Seneviratne conceptualized the review and wrote the first draft. Kalpana Surendranath and Anne Majumdar edited the draft. Mark R. Miller and revised it critically for important intellectual content. All authors have read and approved the final version of this manuscript and agree to be accountable for all aspects of the work in ensuring that questions related to the accuracy or integrity of any part of the work are appropriately investigated and resolved. All persons designated as authors qualify for authorship, and all those who qualify for authorship are listed.

## CONFLICT OF INTEREST

None declared.

## FUNDING INFORMATION

No funding was received for this work.
